# Supplementary material for: Arctic weather variability and connectivity
Source: Nat Commun. 2023 Oct 18;14:6574. doi: 10.1038/s41467-023-42351-x (PMC10584854; doi:10.1038/s41467-023-42351-x)
Supplement: Supplementary file 1 — Supplementary Information [file 41467_2023_42351_MOESM1_ESM.pdf]

Supplementary Information: Arctic weather variability and connectivity

Jun Meng,[1] Jingfang Fan,[2,3,*] Uma S Bhatt,[4,5] and Jürgen Kurths[3,4,5,6]

[1]*School of Science, Beijing University of Posts and Telecommunications, Beijing 100876, China*

[2]*School of Systems Science/Institute of Nonequilibrium Systems, Beijing Normal University, Beijing 100875, China*

[3]*Potsdam Institute for Climate Impact Research, Potsdam 14412, Germany*

[4]*Geophysical Institute, Department of Atmospheric Sciences, University of Alaska Fairbanks, Fairbanks, AK 99775, USA*

[5]*College of Natural Sciences and Mathematics, University of Alaska Fairbanks, Fairbanks, AK 99775, USA*

[6]*Institute of Physics, Humboldt-University, Berlin 10099, Germany*

[*] jingfang@bnu.edu.cn

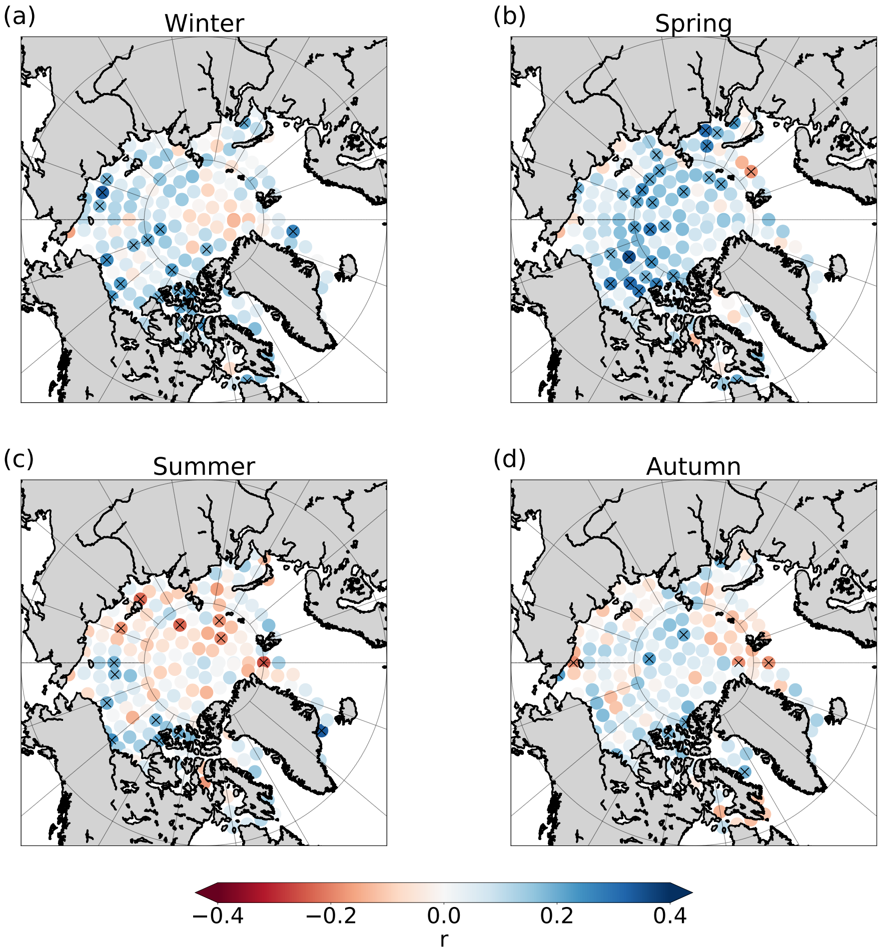

FIG. S1. Heatmaps of correlations between the AO index and the W_{ACF} of the Arctic sea ice. The correlations are shown for different months within distinct seasons: (**a**) Winter, (**b**) Spring, (**c**) Summer, and (**d**) Autumn. This provides insights into the seasonal variations in the relationship between the AO index and W_{ACF}.

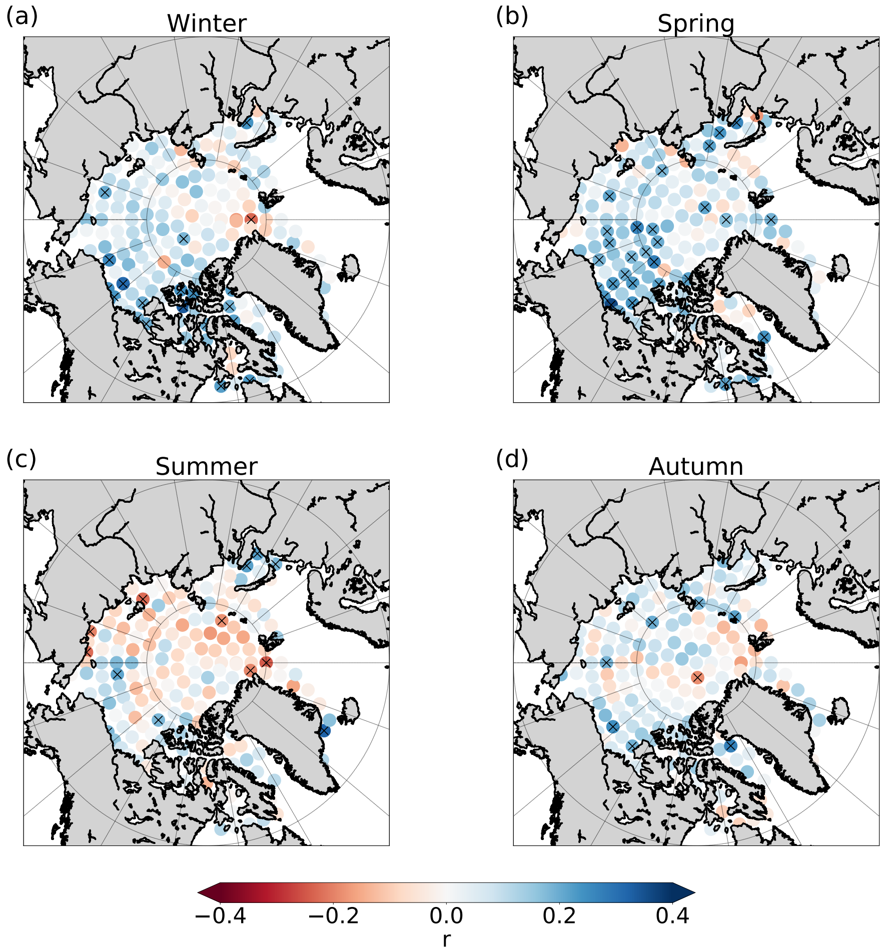

FIG. S2. The same as FIG. S1 but for W_{PS}. Heatmaps of correlations between the AO index and the W_{PS} of the Arctic sea ice. The correlations are shown for different months within distinct seasons: (**a**) Winter, (**b**) Spring, (**c**) Summer, and (**d**) Autumn. This provides insights into the seasonal variations in the relationship between the AO index and W_{PS}.

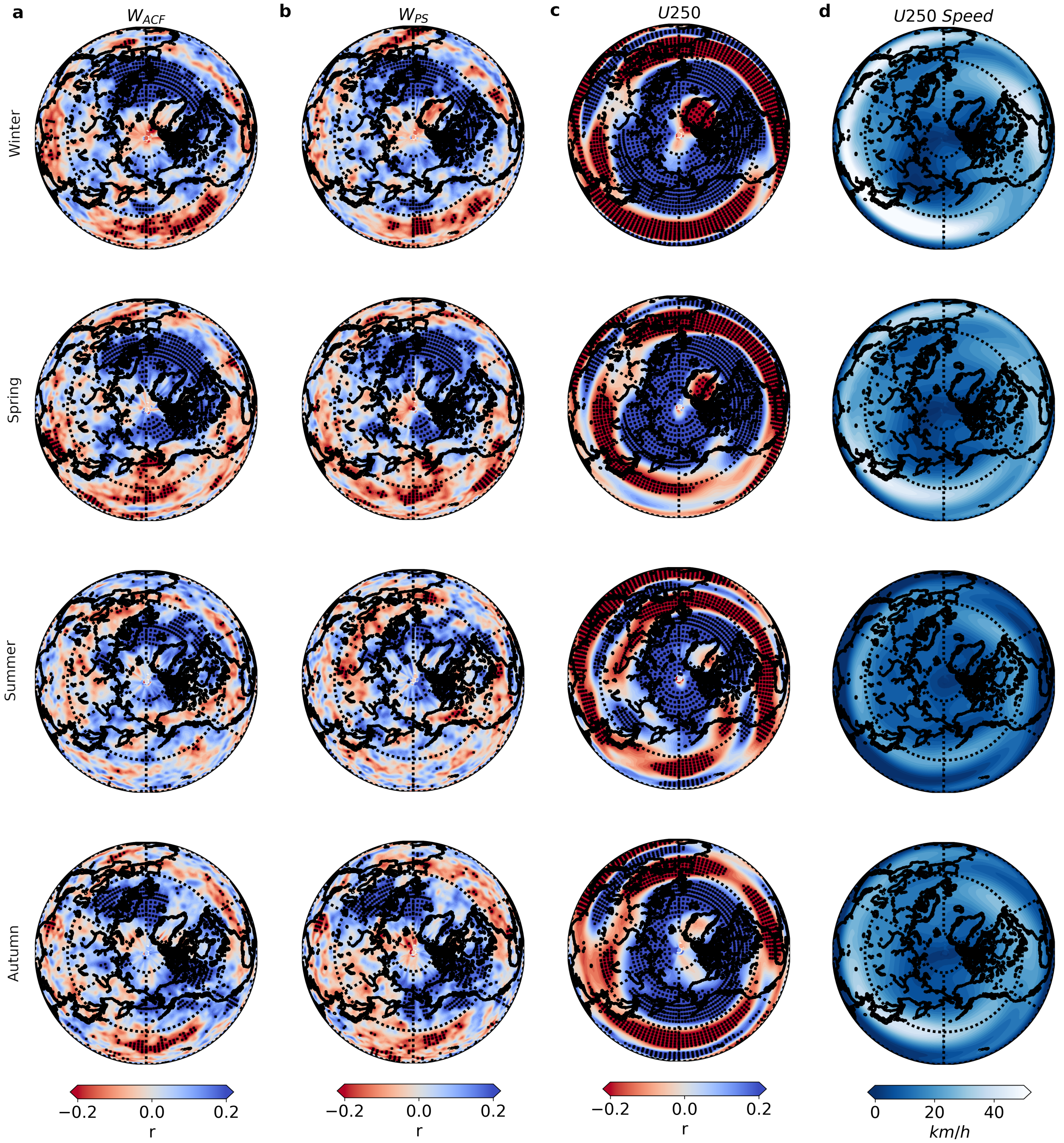

FIG. S3. Visualization of the AO's seasonal influences on Northern Hemisphere local weather variability patterns. (**a**) Heatmaps depicting correlations between the AO and W_{ACF} are shown, considering only values from the same season (i.e., Dec.-Feb for winter, Mar.-May for spring, Jun.-Jul. for summer, and Sep.-Nov. for autumn). (**b**) Analogous to (**a**) but for W_{PS}. (**c**) Similar to (**a**) but for the monthly averaged zonal wind speed at 250hPa pressure level (U250). (**d**) The mean speed of U250 during the period from Jan. 1980 to Dec. 2019. Correlation values with 95% or higher significance are marked by “x”.

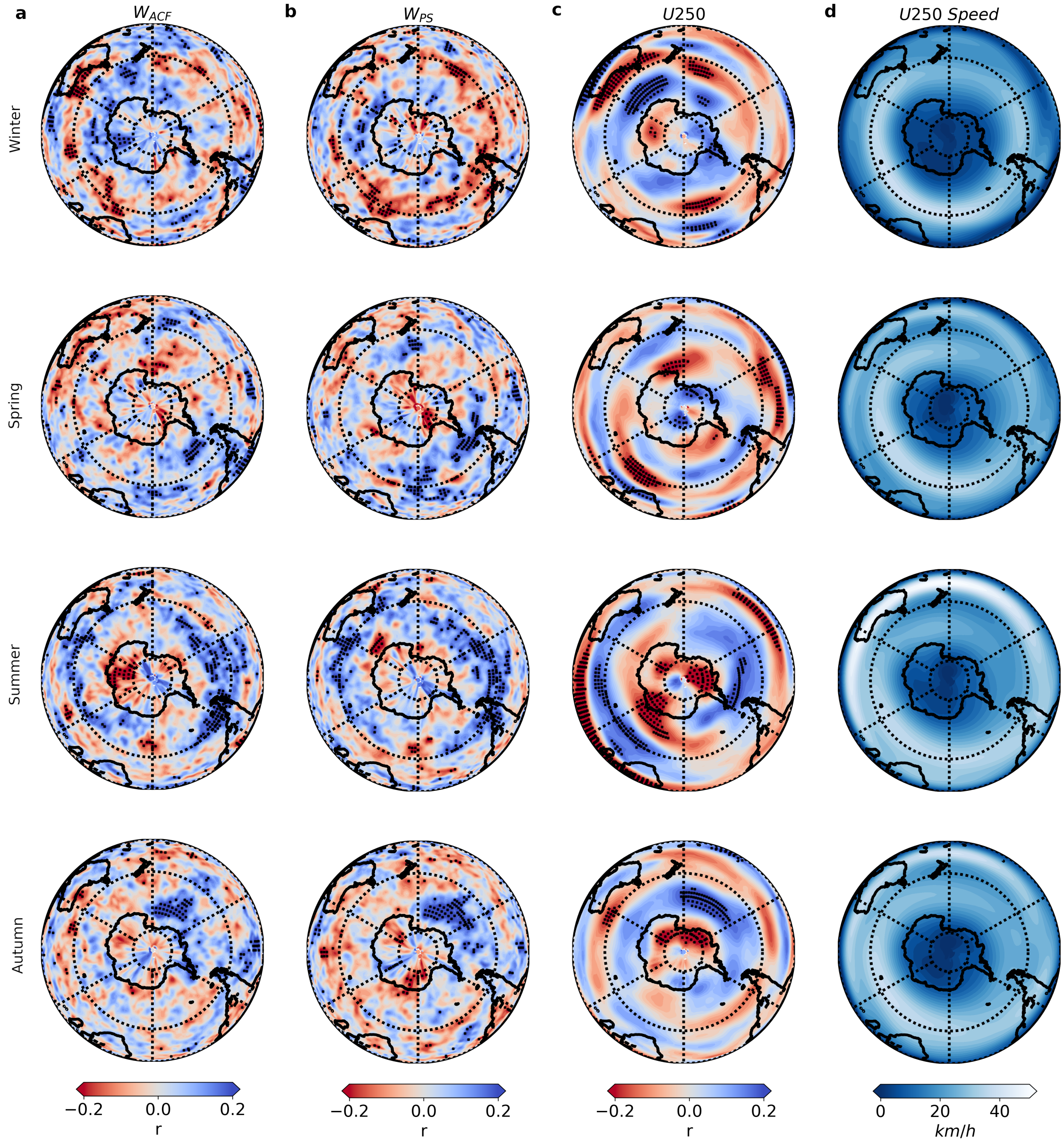

FIG. S4. Analogous to FIG. S3, visualization of the AO's seasonal influences on Southern Hemisphere local weather variability patterns. (**a**) Heatmaps depicting correlations between the AO and W_{ACF} are shown, considering only values from the same season (i.e., Dec.-Feb for winter, Mar.-May for spring, Jun.-Jul. for summer, and Sep.-Nov. for autumn). (**b**) Analogous to (**a**) but for W_{PS}. (**c**) Similar to (**a**) but for the monthly averaged zonal wind speed at 250hPa pressure level (U250). (**d**) The mean speed of U250 during the period from Jan. 1980 to Dec. 2019. Correlation values with 95% or higher significance are marked by "x".

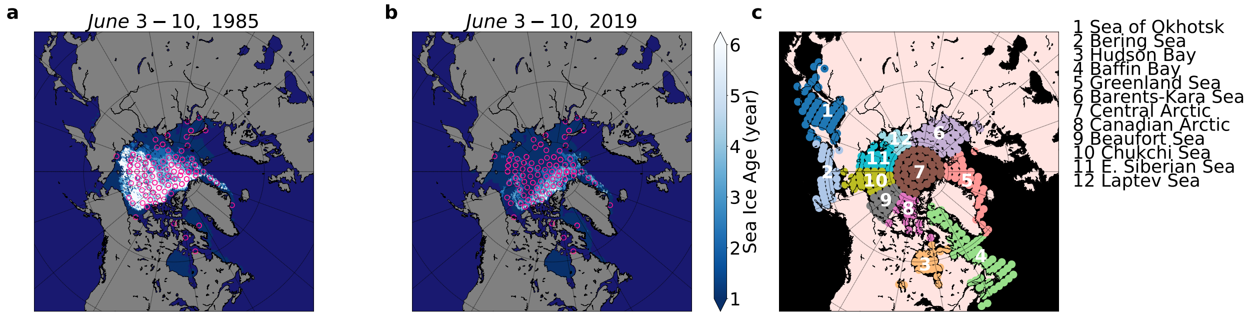

FIG. S5. Decline of the multi-year sea ice cover. (**a**) A sample image displaying sea ice age for the week of Jun. 3-10, 1985 (`https://doi.org/10.5067/UTAV7490FEPB`. [Accessed in Sep. 2021].). (**b**), The same as (**a**) for 2019. Nodes with a significant trend of enhancing W_{ACF} or W_{PS} as shown in Fig. 4 in the main text, are marked by pink circles. (**c**) Depicts the 12 sub-regions of the Arctic. The coloured points indicate grid points with non-zero sea ice cover for at least one day during the years from 1979 to 2019, according to ERA5 datasets.

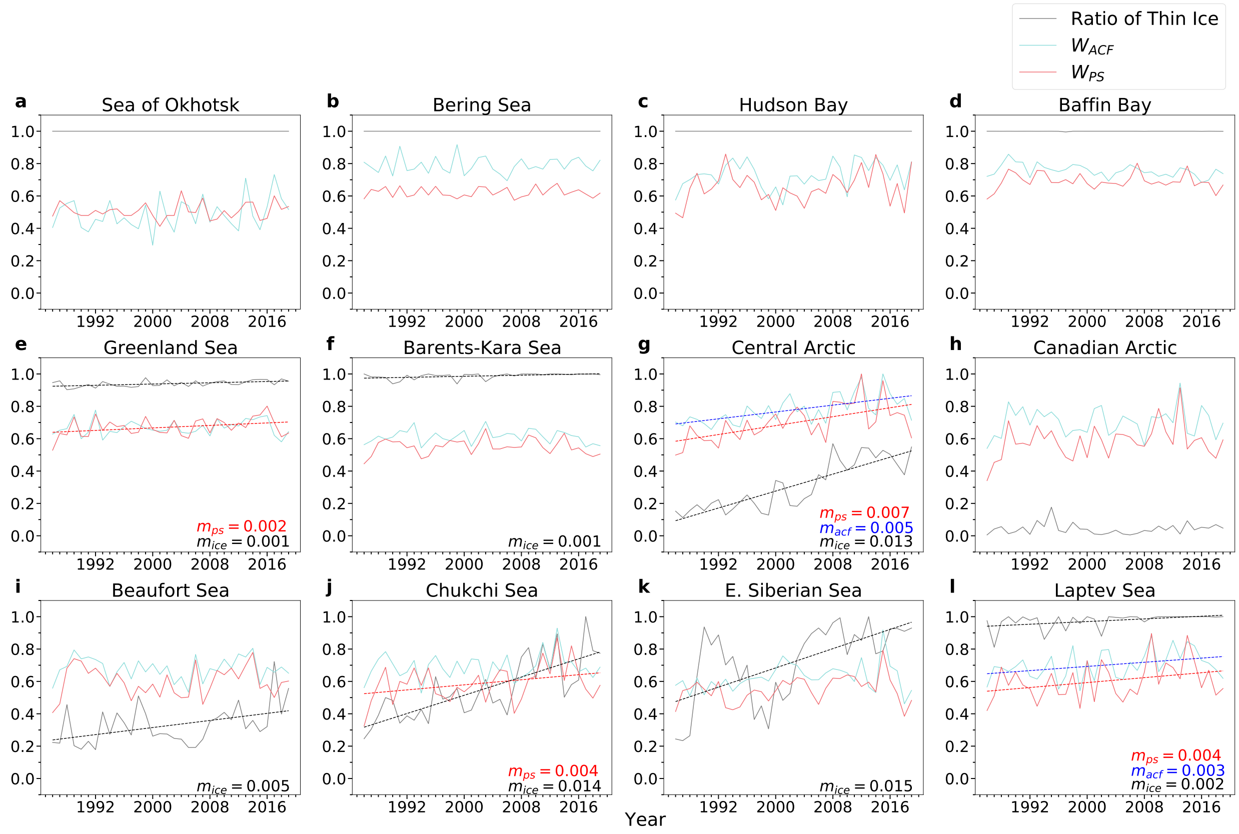

FIG. S6. Panels (**a** - **l**) show the temporal evolution of the ratio of area covered by thin ice (i.e., first-year ice or with ice concentration less than 15%), the averaged W_{ACF} and W_{PS} for 12 subregions shown in FIG. S5 (**c**). The dashed lines are the best-fitting lines with significant trends. The slopes of the significant trends for ice cover, W_{ACF}, and W_{PS} are denoted as m_{ice}, m_{acf}, and m_{ps} respectively.

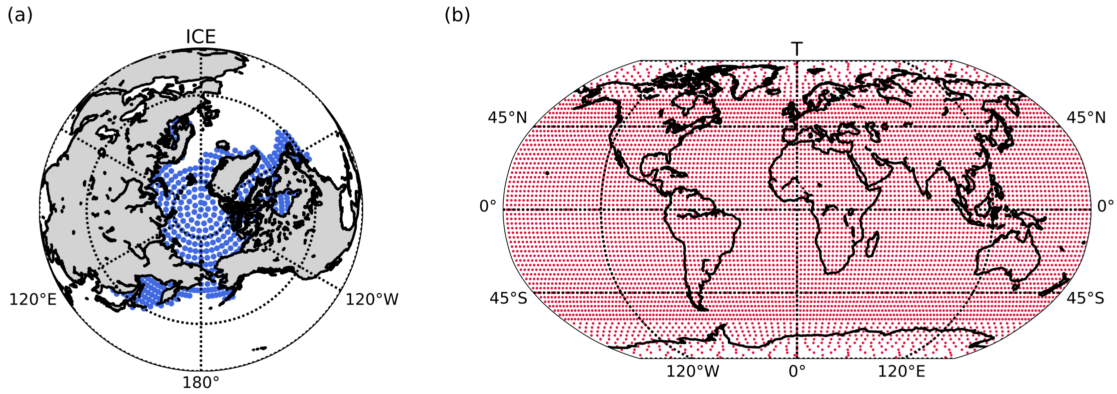

FIG. S7. Diagram for the climate network. (**a**) 377 Arctic nodes with non-zero sea ice cover for at least one day during the years from 1979 to 2019, according to ERA5 datasets. (**b**) 8040 grid points of the ERA5 850 hPa daily air temperature field that approximately equally covering the globe.

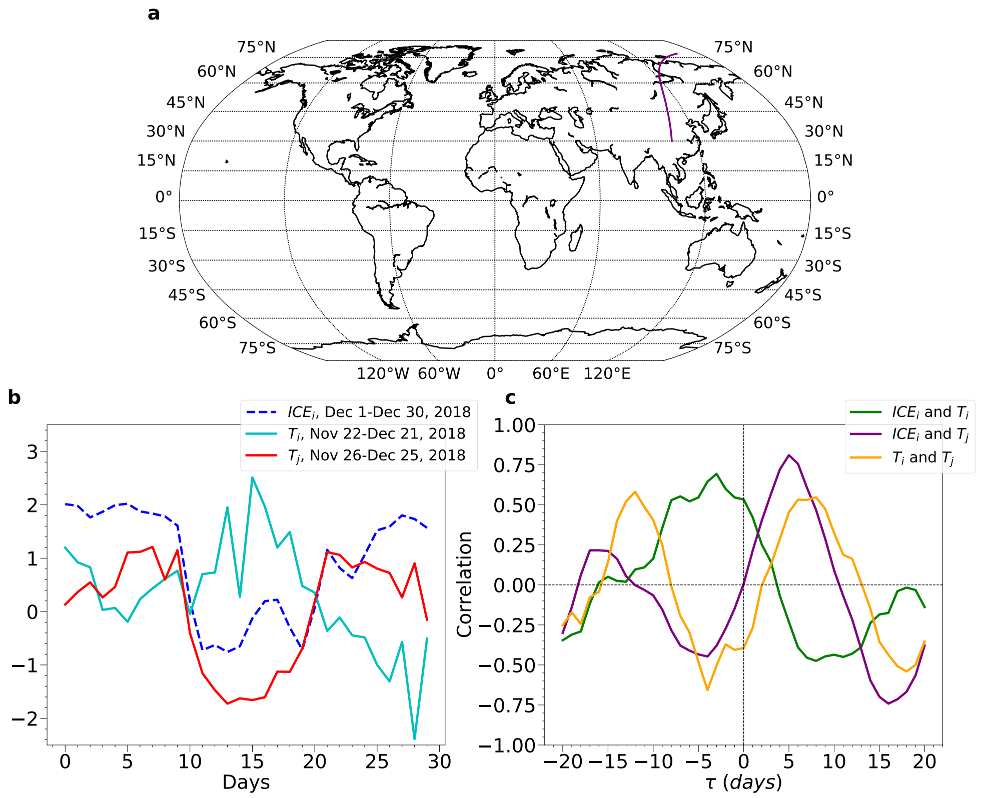

FIG. S8. An example of teleconnection between an Arctic node i (77.5°N, 160°E) and a global node j (30°N, 105°E) located in Southwest China. (**a**) The link connects node i and node j is marked by purple on the map. (**b**) Time series for the sea ice cover I_i and the 850hPa air temperature T_i, T_j. The time span of each time series is shifted to the dates that the correlation function obtains its maximum, the dates in the legend indicate time span of the corresponding time series, all time series are normalized by dividing one standard deviation. (**c**) The correlation functions among time series of the sea ice cover data and temperature data for node i, and the temperature data for node j.

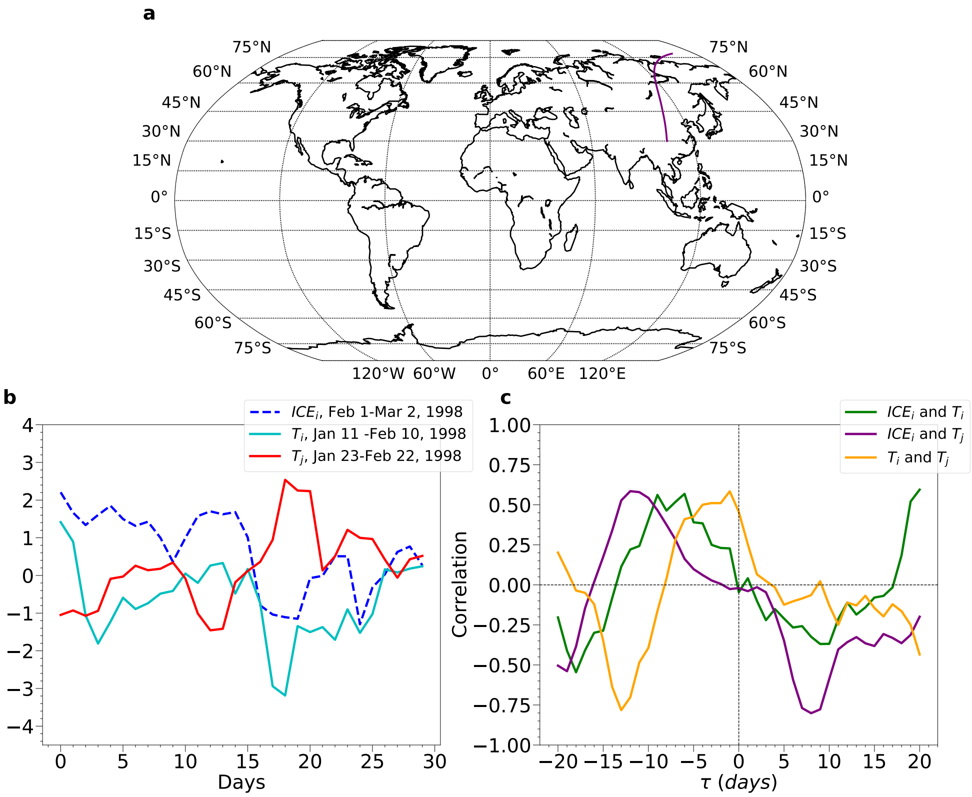

FIG. S9. The same as FIG. S8 but for another month. (**a**) The link connects node i and node j is marked by purple on the map. (**b**) Time series for the sea ice cover I_i and the 850hPa air temperature T_i, T_j. The time span of each time series is shifted to the dates that the correlation function obtains its maximum, the dates in the legend indicate time span of the corresponding time series, all time series are normalized by dividing one standard deviation. (**c**) The correlation functions among time series of the sea ice cover data and temperature data for node i, and the temperature data for node j.

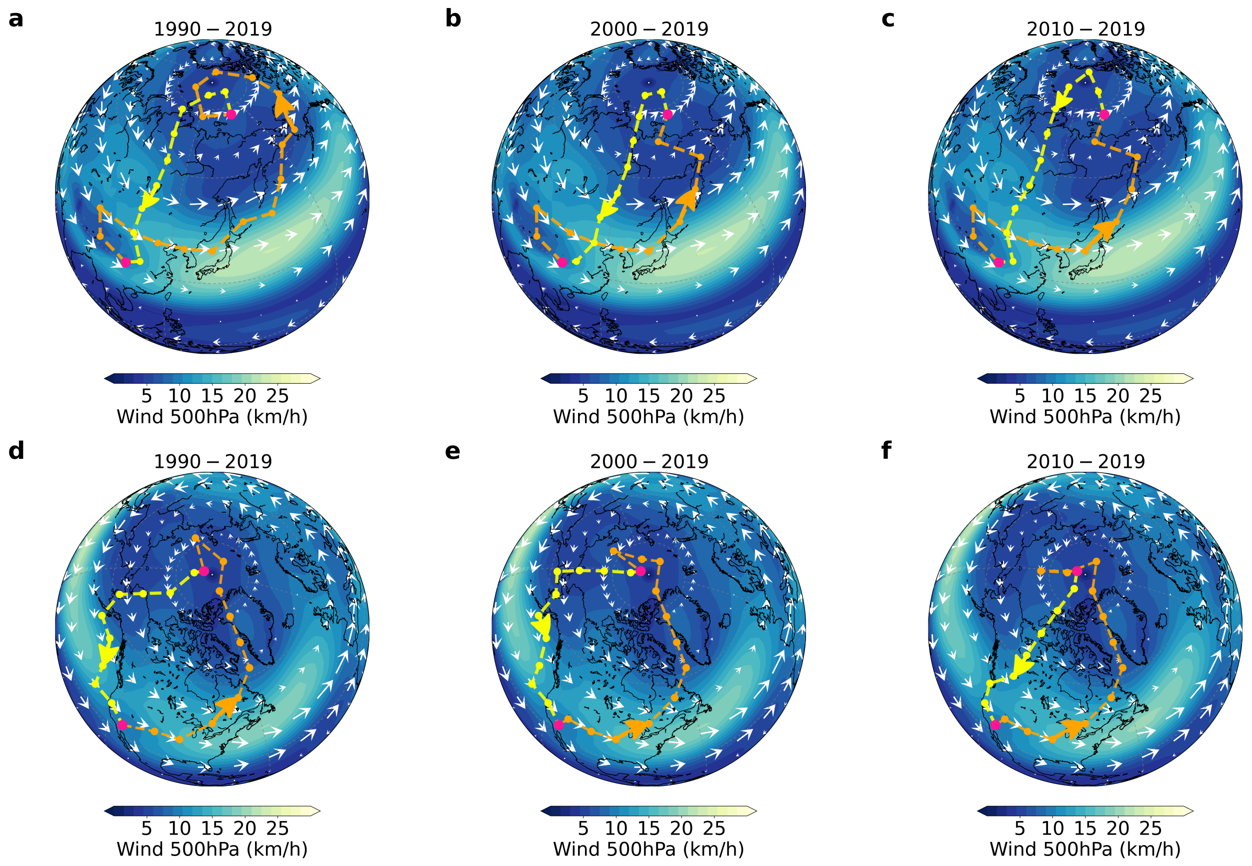

FIG. S10. Panels (**a-c**), The shortest path method identifies propagation pathways in climate networks constructed from global air temperature data over the most recent 30, 20 and 10 years. These pathways are between a node in Southwest China (30°N, 105°E) and an Arctic node (77.5°N, 160°E). Panels (**d-e**), Similar to Panels (**a-c**), but for another teleconnection between California (35°N, 115°W) and Arctic (87.5°N, 165°W). The visualization of these pathways features background colors representing the averaged wind speed (500 hPa), with white arrows indicating the prevailing wind direction over the corresponding periods.

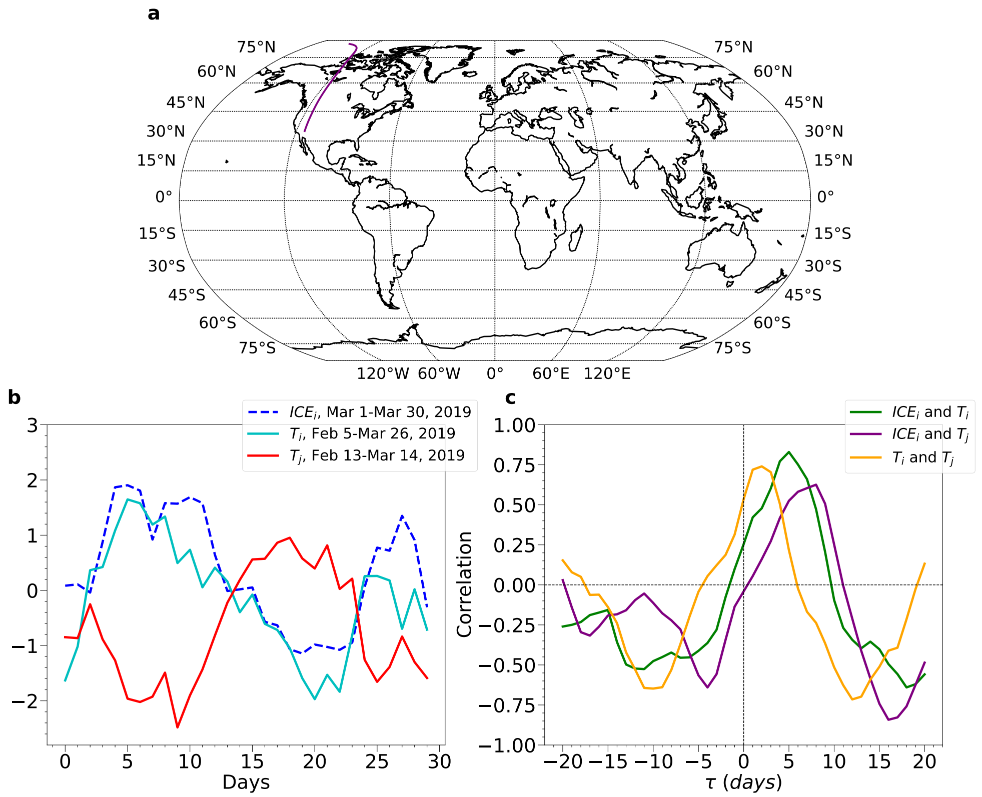

FIG. S11. The same as FIG. S8 but for another typical link connecting an Arctic node (87.5°N, 165°W) and a global node (35°N, 115°W) located in California. (**a**) The link connects node i and node j is marked by purple on the map. (**b**) Time series for the sea ice cover I_i and the 850hPa air temperature T_i, T_j. The time span of each time series is shifted to the dates that the correlation function obtains its maximum, the dates in the legend indicate time span of the corresponding time series, all time series are normalized by dividing one standard deviation. (**c**) The correlation functions among time series of the sea ice cover data and temperature data for node i, and the temperature data for node j.

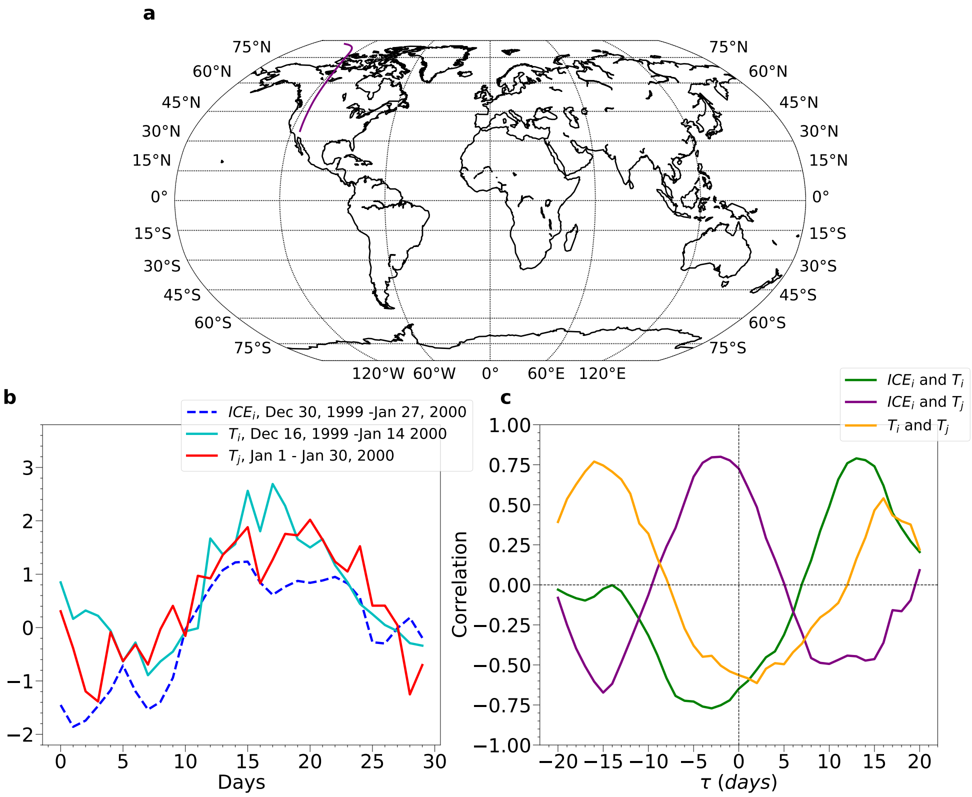

FIG. S12. The same as FIG. S11 but for another month. (**a**) The link connects node i and node j is marked by purple on the map. (**b**) Time series for the sea ice cover I_i and the 850hPa air temperature T_i, T_j. The time span of each time series is shifted to the dates that the correlation function obtains its maximum, the dates in the legend indicate time span of the corresponding time series, all time series are normalized by dividing one standard deviation. (**c**) The correlation functions among time series of the sea ice cover data and temperature data for node i, and the temperature data for node j.

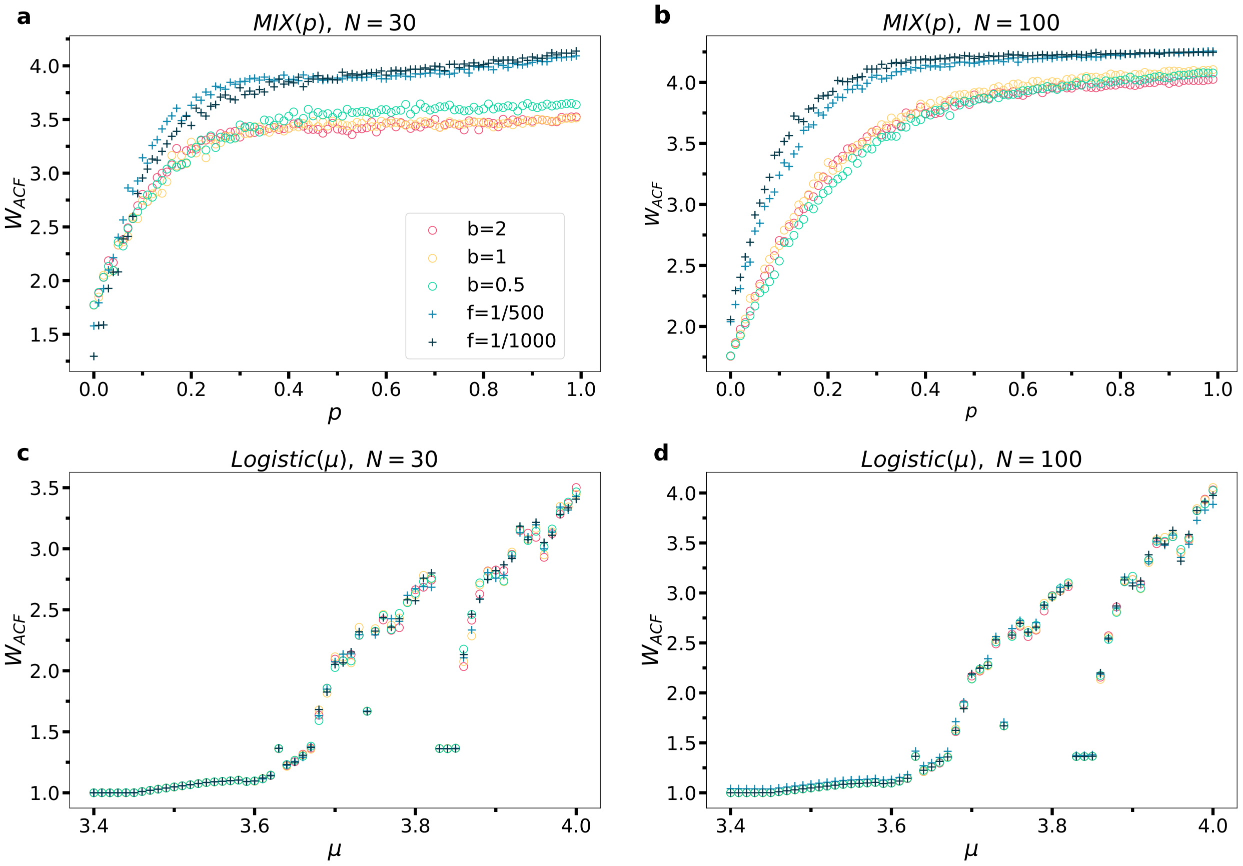

FIG. S13. An intuitive demonstration of the effectiveness of W_{ACF} in quantifying the variability of non-stationary data. The W_{ACF} values are calculated for the MIX(p) model (**a-b**) and Logistic models (**c-d**), which exhibit strong monotonous trends of the form $1000i^b$ with varying power values ($b = 2, 1$, and 0.5), as well as superimposed oscillatory trends of the form $10sin(2\pi if)$ with different frequencies ($f = 1/500, 1/10000$). The W_{ACF} consistently changes with parameters (p and μ) that control the chaotic behavior of the data. N represents the length of the time series, and each W_{ACF} value is obtained by averaging over 100 realizations.
